# Peptidyl arginine deiminase 2 (*Padi2*) is expressed in Sertoli cells in a specific manner and regulated by SOX9 during testicular development

**DOI:** 10.1038/s41598-018-31376-8

**Published:** 2018-09-05

**Authors:** Atsumi Tsuji-Hosokawa, Kenichi Kashimada, Tomoko Kato, Yuya Ogawa, Risa Nomura, Kei Takasawa, Rowena Lavery, Andrea Coschiera, David Schlessinger, Vincent R. Harley, Shuji Takada, Tomohiro Morio

**Affiliations:** 1 Department of Pediatrics and Developmental Biology, Tokyo Medical and Dental University, Bunkyo-ku, Tokyo, 113-8510 Japan; 2 Department of Systems BioMedicine, National Research Institute for Child Health and Development, Setagaya-ku, Tokyo, 157-8535 Japan; 3 Center for Endocrinology and Metabolism, Hudson Institute of Medical Research, Clayton, VIC 3168 Australia; 4 IRP Laboratory of Genetics, NIH Biomedical Research Center National Institute on Aging, Baltimore, 21224 MD USA

## Abstract

Peptidyl arginine deiminases (PADIs) are enzymes that change the charge of proteins through citrullination. We recently found *Padi2* was expressed exclusively in fetal Sertoli cells. In this study, we analyzed the transcriptional regulation of *Padi2* and the role of PADI2 in testicular development. We showed SOX9 positively regulated *Padi2* transcription and FOXL2 antagonized it in TM3 cells, a model of Sertoli cells. The responsive region to SOX9 and FOXL2 was identified within the *Padi2* sequence by reporter assay. In fetal testes from *Sox9* knockout (AMH-Cre:*Sox9*^*flox/flox*^) mice, *Padi2* expression was greatly reduced, indicating SOX9 regulates *Padi2 in vivo*. *In vitro* analysis using siRNA suggested PADI2 modified transcriptional regulation by SOX9. However, *Padi2*^−/−^ XY mice were fertile and showed no apparent reproductive anomalies. Although, PADI2 is known as an epigenetic transcriptional regulator through H3 citrullination, no significant difference in H3 citrullination between wildtype and *Padi2*^−/−^ XY gonads was observed. These results suggest *Padi2* is a novel gene involved in testis development that is specifically expressed in Sertoli cells through the regulation by SOX9 and FOXL2 and PADI2 supports regulation of target genes by SOX9. Analysis of the *Padi2*^−/−^ XY phenotype suggested a redundant factor compensated for PADI2 function in testicular development.

## Introduction

SRY-box 9 (*Sox9*) is the major target of a male-determining gene on the Y chromosome, SRY, and its product SOX9 specifies the differentiation and function of Sertoli cells from somatic cell precursors, which then orchestrate the development and maintenance of other testicular cell types. By using genetically modified mice, *Sox9* has been demonstrated to be the determinant and hallmark of Sertoli cell differentiation, i.e., SOX9 has the capacity to drive testis differentiation in the absence of *Sry* in transgenic XX embryos^1^, and deletion of *Sox9* prior to sex determination leads to XY sex reversal^2^.

SOX9 is a member of a highly conserved family of transcription factors defined by their similarity to the high mobility group DNA-binding domain of SRY, and to date, several target genes, such as *Fgf9*, *Amh*, *Ptgds*, and *Cyp26b1*, have been identified^3–6^. Further, recent bioinformatics data analyses, including chromatin immunoprecipitation (ChIP) followed by microarray hybridization (ChIP-chip) or high-throughput sequencing (ChIP-seq), revealed that SOX9 recognizes numerous genes^7–9^.

These data helped to produce an outline of the transcriptional network of SOX9, but the precise mechanisms of Sertoli cell differentiation and their maintenance remain mostly unclear.

Recently, we identified a molecule, peptidyl arginine deiminase, protein-2 (PADI2), that is expressed specifically in Sertoli cells during testicular development. PADI2 has been reported to convert the amino acid arginine in a protein into the amino acid citrulline^10^. Citrullination can occur in any protein, including histone H3 proteins, suggesting that PADI2 mediates gene regulation through epigenetic mechanisms^11^. In order to identify the role of PADI2 in testicular development, we conducted *in vitro* and *in vivo* analyses, including generating *Padi2* knock-out mice.

## Results

### *Padi2* was exclusively expressed in Sertoli Cells in fetal developing testes

**Figure 1:**
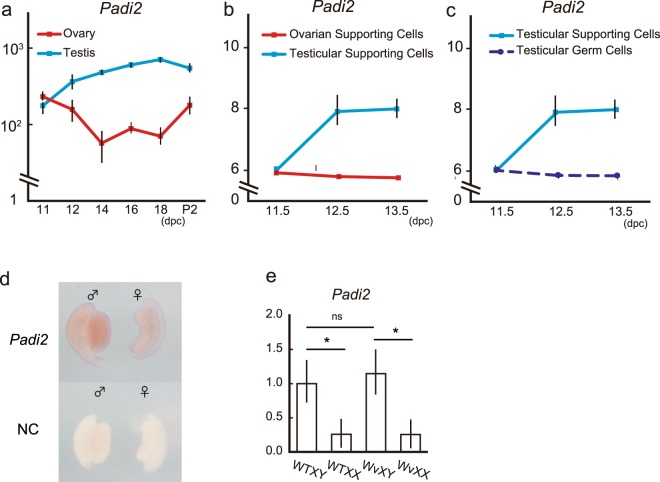
Padi2 was exclusively expressed in Sertoli Cells in fetal developing testes. (**a**–**c**) Expression profile of *Padi2* in fetal mice gonads obtained from the GEO Profiles database. During fetal period, testicular supporting cells expressed *Padi2*. P2, postnatal day 2. Data accessible at NCBI GEO database^12^, accession GSE27715^13^, GSE4818, and GSE5334; Gaido K, Lehmann K *et al*., 2006. (**d**) Whole mount *in situ* hybridization of at 13.5dpc gonads. *Padi2* expression was limited to testicular cord of male gonads. (**e**) *Padi2* expression of gonad in WT and Kit^*Wv/Wv*^ mice at 13.5dpc relative to *ActB* was quantified by qRT-PCR analysis. *Padi*2 expression in XY gonads was maintained in Kit^*Wv/Wv*^ XY mice. Mean ± SD of three biologically independent experiments performed in triplicate was shown. Asterisks indicate level of statistical significance (*P < 0.05; NS: not significant). Statistical significance was determined using one-way ANOVA followed by a Tukey-Kramer post hoc test.

Based on previously reported microarray data, we found that *Padi2* is expressed in a testes-specific manner over the prenatal period (Fig. 1a) and transcriptional profiling of somatic and germ cells suggested that testicular supporting cells expressed *Padi2* (Fig. 1b,c) (data accessible at NCBI GEO database^12^, accession GSE27715^13^, GSE4818, and GSE5334; Gaido K, Lehmann K *et al*., 2006). In order to confirm these data, we employed whole mount *in situ* analysis and RT-PCR analysis of *Kit*^*Wv/Wv*^ mouse gonads. *Padi2* was expressed in the testicular cord (Fig. 1d), and its expression was limited to somatic cells because *Padi2* expression was not reduced in *Kit*^*Wv/Wv*^ mice, in which germ cells are absent (Fig. 1e). These results suggested that *Padi2* is exclusively expressed in testicular supporting cells, i.e., Sertoli cells.

### Antagonistic regulation of *Padi2* by SOX9 and FOXL2 *in vitro*, but not *in vivo*

**Figure 2:**
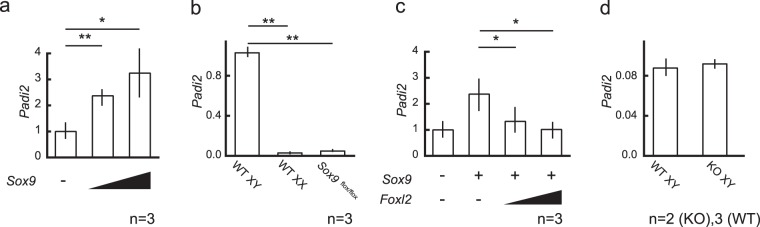
Antagonistic regulation of Padi2 by SOX9 and FOXL2 *in vitro*, but not *in vivo*. (**a**) Introduction of *Sox9* into TM3 cells significantly increased *Padi2* expression. (**b**) *Padi2* expression in XY gonads of AMH-Cre:*Sox9*^*flox/flox*^ mice at 13.5dpc was remarkably reduced at almost similar level of wildtype XX gonads. (**c**) The transcriptionally positive regulation of *Padi2* by SOX9 was attenuated by ovarian molecule, FOXL2 in TM3 cells. (**d**) *Padi2* expression in XY gonads of *Foxl2*-null mice at 13.5 dpc. *Padi2* expression was not changed in Foxl2 null mice. The mean and SD of three biological replicates, except Foxl2 null mice, measured in triplicate was calculated. The data of *Foxl2*-null mice was from two biological replicate samples and mean value was calculated. Asterisks indicate level of statistical significance (*P < 0.05; **P < 0.01). Unpaired Student’s *t*-test was used to demonstrate statistically significant difference between the given sample and the control.

The temporal expression pattern of *Padi2* suggested that the gene would be a target of the determinant of Sertoli cell differentiation, SOX9, and we carried out *in vitro* analyses using TM3 cells, a model of Sertoli cells^14^. Consistently, *Sox9* introduction into TM3 cells transcriptionally upregulated endogenous *Padi2* expression in a dose-dependent manner (Fig. 2a).

In order to investigate *Padi2* regulation by SOX9 *in vivo*, we employed the XY gonads of AMH-Cre:*Sox9*^*flox/flox*^ mice. The XY gonads of AMH-Cre:*Sox9*^*flox/flox*^ mice are phenotypically testes, and *Sox9* expression is markedly reduced at 13.5 dpc, after the time of sex determination, and absent by 14.5 dpc, enabling a more specific analysis of SOX9 action^15^. In consistent with data from TM3 cells, in AMH-Cre:*Sox9*^*flox/flox*^ XY gonads at 13.5 dpc, *Padi2* expression was dramatically suppressed, and its expression was almost abolished (Fig. 2b).

On the other hand, the positive regulation of *Padi2* by SOX9 was attenuated by the ovarian molecule FOXL2 in TM3 cells (Fig. 2c). However, in *Foxl2*-null mice, *Padi2* expression in XY gonads was not changed (Fig. 2d. In developing testes, *Padi2* expression mostly depends on SOX9 (Fig. 2b) as observed in AMH-Cre:*Sox9*^*flox/flox*^ XY gonads, and suppression by FOXL2 is not required *in vivo*.

### SOX9 and FOXL2 transcriptionally regulated *Padi2* expression through a sequence on intron 1

**Figure 3:**
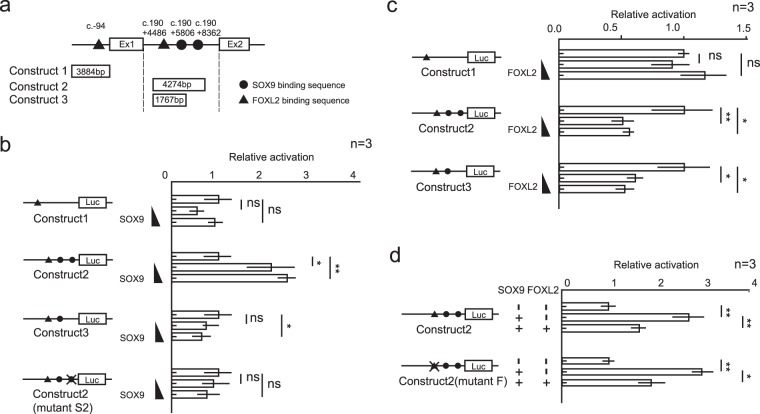
SOX9 and FOXL2 transcriptionally regulated Padi2 expression through a sequence on intron 1. (**a**) Schematic figure of the structure of *Padi2* from the upstream of 5′ UTR to exon2 and constructs used for reporter assay. Closed circle and black triangle indicate SOX9 and FOXL2 possible binding site, respectively. (**b**–**d**) Reporter assay of the five constructs, construct #1~#3 and their mutated constructs, was performed by introducing *Sox9* and/or *Foxl2* into HEK293T cells. 50–75 ng of pSGSox9 and/or 25–75 ng of pcDN3Foxl2 was transfected to the 1.5 × 10^5^ cells. The mean and SD of three biological replicates measured in triplicate was calculated. Asterisks indicate level of statistical significance (*P < 0.05; **P < 0.01, ns, not significant). Unpaired Student’s *t*-test was used to demonstrate statistically significant difference between the given sample and the control for (**a**–**c**). For (**d**) one-way ANOVA followed by a Tukey-Kramer post hoc test was used for analysis.

In order to elucidate the precise mechanisms of the regulation by SOX9 and FOXL2, we examined the sequence upstream of the 5ʹ-UTR and all introns of *Padi2*. Upstream of the gene, one possible biding sequence for FOXL2 (c.−94) was identified, while one possible binding sequence for FOXL2 (c.190 + 4486) and two for SOX9 (c.190 + 5806, c.190 + 8362) were identified (Fig. 3a) in the first intron. We employed three reporter constructs, #1, #2, and #3, that harbor possible binding sequences, as shown in Fig. 3a, and conducted a series of *in vitro* co-transfection reporter assays in HEK293T cells, which do not express endogenous *Sox9* and *Foxl2*.

The reporters of construct #2, but not of constructs #1 and #3, were activated by *Sox9* introduction in a dose-dependent manner (Fig. 3b), suggesting that c.190 + 8362 was responsible for the transactivation by SOX9. Consistently, by introducing mutations into the sequence, c.190 + 8362, the response of construct #2 was completely abolished (Fig. 3b).

Repression by FOXL2 was observed in constructs #2 and #3 but not in #1 (Fig. 3c). As shown in Fig. 2, FOXL2 cancelled the positive regulation of SOX9 for construct #2 (Fig. 3d). However, the suppressive effects were also observed in the reporter with a mutated possible biding site of FOXL2, construct #2 (mutant F) (Fig. 3d). On the other hand, construct #2 was suppressed by FOXL2 in the absence of SOX9 (Fig. 3c), suggesting that FOXL2 suppressed the transcription of *Padi2* by binding to an unknown element of intron 1 rather than by interfering with the access of SOX9 to its binding element.

### Transcriptional modification of the SOX9 target genes by PADI2 *in vitro*

Next, we examined the possible role of *Padi2* in testicular development *in vitro*. PADI2 is a peptidyl arginine deiminase that converts the amino acid arginine into citrulline, and it has been suggested to modify transcriptional activity by an epigenetic mechanism, the citrullination of the histone protein H3^11^. Considering that *Padi2* is downstream of SOX9, one possible explanation for the role of PADI2 is that it is a modulator of SOX9 action itself.

**Figure 4:**
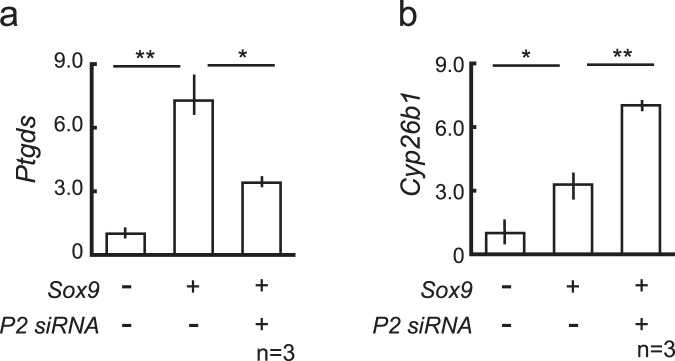
Transcriptional modification of the SOX9 target genes by PADI2 *in vitro*. The SOX9 target genes, *Ptgds* (**a**) and *Cyp26b1* (**b**) mRNA levels in TM3 cells (3.0 × 10^5^/well) transfected with pSGSox9 (2–4 µg) and/or siRNA of *Padi2* (50 µM). All data sets represent qRT-PCR analysis of *Ptgds* or *Cyp26b1* mRNA expression relative to *Gapdh* (means ± SD of 3 biologically independent experiments performed in triplicate). Asterisks indicate level of statistical significance (*P < 0.05; **P < 0.01). Statistical significance was determined using one-way ANOVA followed by a Tukey-Kramer post hoc test.

In order to verify this hypothesis, we knocked down *Padi2* expression by siRNA and co-transfected SOX9 and then examined the expression levels of the target genes of SOX9. For the *Ptgds* gene, transcriptional activation by SOX9 was attenuated by knocking down *Padi2* (Fig. 4a), while the inhibition of *Padi2* expression additionally up-regulated the expression of the *Cyp26b1* gene in TM3 cells (Fig. 4b), suggesting that PADI2 modifies SOX9 transcriptional regulation.

### *Padi2*^−/−^ XY mice did not exhibit an obvious testicular phenotype, including fertility

**Figure 5:**
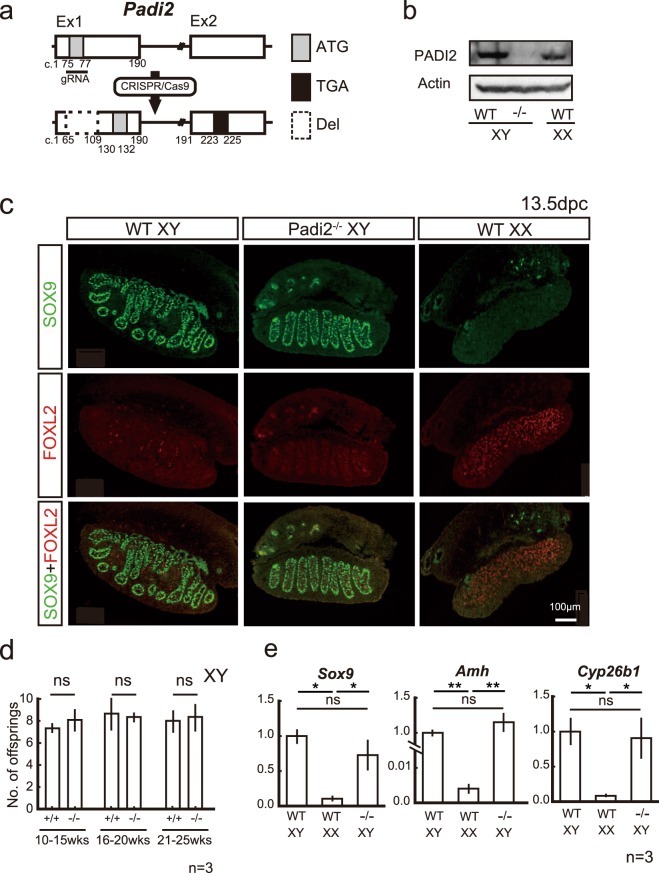
Padi2 KO XY mice did not exhibit obvious testicular phenotype with normal fertility. (**a**) Schematic representation of the strategy to generate *Padi2*^−/−^ mice using CRISPR/Cas9 system. A guide RNA (gRNA) was designed to delete a part of exon 1 including the first ATG (c.75–77), and a sequence of 45 bp from c.65 to c.109 (dotted lined box) was deleted, resulting in a predicted variant of PADI2 from c. 130 in exon1 to c.225 in exon2. (**b**) In *Padi2*^−/−^ mice, the expression of PADI2 was abolished in adult mice brain. (**c**) *Padi2* KO XY mice did not exhibit obvious testicular phenotype in immunofluorescence analysis. (**d**) The number of offspring was identical with that of the wild type from young adult (10–15 weeks of age) to matured adult mice (21–25 weeks of age). Three male mice for each group were mated with C57BL/6J wild type female mice and examined. The graphs indicate means and SD of each litter size. (**e**) mRNA levels of SOX9 target genes in *Padi2*^−/−^ mice at 13.5 dpc. All data sets represent qRT-PCR analysis of *Sox9*, *Amh* or *Cyp26b1* mRNA expression relative to *ActB* (means ± SD of 3 biologically independent experiments performed in triplicate). Asterisks indicate level of statistical significance (*P < 0.05; **P < 0.01, ns, not significant). Unpaired Student’s *t*-test was used to analyze significant difference for (**d**). One-way ANOVA followed by a Tukey-Kramer post hoc test was used for analysis of (**e**). Full-length blots are presented in Supplementary Fig. S1a.

To elucidate the role of PADI2 during testicular development *in vivo*, we generated *Padi2*^−/−^ mice using the CRISPR/Cas9 system. By targeting exon 1 (Fig. 5a), *Padi2*^−/−^ mice were successfully established (Fig. 5b). *Padi2*^−/−^ XY mice had normal male external genitalia (data not shown). Histological analyses revealed that *Padi2*^−/−^ XY gonads exhibited normal testicular cord formation. SOX9 was expressed in wildtype testis, and the expression of the ovarian marker *FOXL2* was not detected (Fig. 5c). *Padi2*^−/−^ mice were fertile, and the number of offspring was identical with that of the wildtype (Fig. 5d). No significant difference was observed in the expression of SOX9 and its target genes between wildtype and knock-out mice (Fig. 5e).

### H3 citrullination in developing testes was not affected in *Padi2*^−/−^ mice

**Figure 6:**
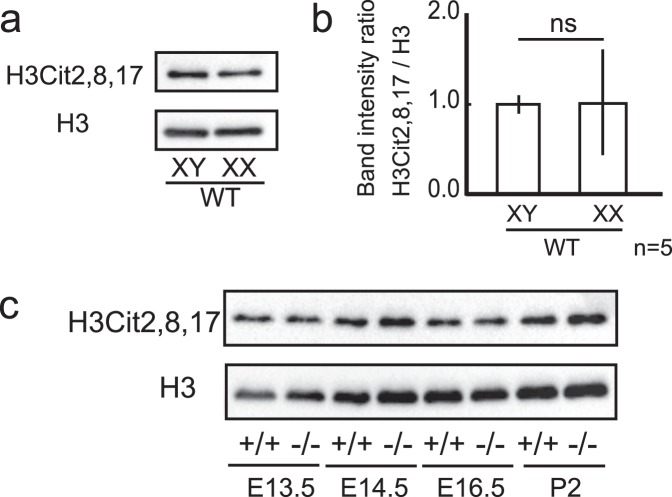
H3 Citrullination in developing testes was not affected in Padi2 KO mice. (**a**,**b**) Citrullination of H3 in male and female mice gonads at postnatal days (P2) was analyzed by immunoblotting (**a**), and the intensity of H3 citrullination from 5 biologically independent samples was quantified relative to H3 (means ± SD) (**b**). Unpaired Student’s *t*-test was used to analyze significant difference. ns: not significant. (**c**) Immunoblotting analysis revealed, in XY gonads of *Padi2*^−/−^ mice, the level of citrullination, H3Cit2, 8, 17, was not attenuated compared to that of wild type mice. Full-length blots are presented in Supplementary Fig. S1b–g.

It has been reported that PADI2 is involved in an epigenetic mechanism, the citrullination of H3, and we examined the citrullination of H3 in developing gonads. In wildtype XX and XY gonads, the level of citrullination, H3Cit2, 8, 17, was identical (Fig. 6a,b), and in the XY gonads of *Padi2*^−/−^ mice, the level of citrullination, H3Cit2, 8, 17, was not attenuated compared to that of the wildtype (Fig. 6c). These findings suggested that in fetal developing testes, citrullination of H3 was not dependent on PADI2.

## Discussion

Our present study revealed that *Padi2* is specifically expressed in Sertoli cells and transcriptionally activated by the testicular determining factor SOX9. Previously, PADI2 function has been reported in other biological contexts, such as H3 citrullination during breast cancer oncogenesis^16–18^ the estrus cycle in the uterus or in gonadotrope cells^11,19,20^ peptidyl citrullination during colon cancer oncogenesis^21,22^ and inflammation inducing multiple sclerosis and skin neoplasia^23–25^. Regarding embryonic development, other isoforms of PADI have been suggested to be involved: PADI4 was found to regulate pluripotency via H1 citrullination^26^ and PADI6 was found to function during zygotic genome activation and be related to female infertility^27^. Our study is the first report to suggest a potential role of PADI2 in organ development.

One of the possible roles of PADI2 in testicular development is epigenetic function through the citrullination of histone H3. The citrulline amino acid residues are only produced through post-translational modifications catalyzed by PADI, which is encoded by the *Padi* gene^28^. Our study did not identify a significant difference in citrullinated H3 between wildtype mice and *Padi2*^−/−^ mice, suggesting that the citrullination of histone H3 is independent of PADI2 during gonadal development. To date, only two subtypes of PADI, PADI2 and PADI4, are considered to be involved in H3 citrullination. Although we did not examine the citrullination of H3 in the testes of *Padi4* knock-out mice, *Padi4* is not likely to contribute to the citrullination of H3 because of its low expression. Our data suggested the possibility that unknown mechanisms contributed to the citrullination of H3 during gonadal development.

Although the data from *Padi2* KO mice suggested that the citrullination of histone H3 is independent of PADI2 during gonadal development, *Padi2* affected the transcriptional regulation of SOX9 in TM3 cells. One possible explanation for this finding could be the citrullination of other proteins that affect SOX9 function. A homologue of PADI2, PADI4, has been reported to regulate co-factor interaction with transcriptional factors by citrullination, affecting transcriptional regulation^29,30^.

A specific high-affinity FOXL2 binding element (FLRE) has been reported, 5′-GT(C/G)AAGG-3′^31^, and we identified two regions within the promoter and intron 1 in the *Padi2* gene. However, our reporter assay revealed that the region was not responsible for the suppressive effect of FOXL2. The series of experiments designed to determine the role of FOXL2 in gonadotropin hormone regulation revealed that FOXL2 binds to sequences other than FLRE. In this regulation, the FOXL2 binding sequences do not seem to be strictly conserved and could depend on the biological context, including interaction with SMADs and other proteins^32,33^. Given these data, we speculate that FOXL2 binds to intron 1 of *Padi2* through an unidentified sequence other than FLRE.

It was intriguing that *Padi2* expression was markedly repressed in AMH-Cre:*SOX9*^*flox/flox*^ mice. This result suggests that during testicular development, the transcriptional regulation of *Padi2* would escape the redundant function of other SOXs, such as SOX8 and SOX10. Although testicular determination is solely SOX9 dependent, SOX9 positively regulates testicular genes, such as *Amh* and *Dhh*, together with SOX8 and SOX10 (two SOX-E subfamily members) after sex determination and maintains testicular development^15,34–36^. We presume that the redundant regulation of testicular-specific genes by SOX E subfamily members could be balanced by the biological context. The essential genes for testicular development could be redundantly regulated by SOX E subfamily members because the testicular phenotype of AMH-Cre:*SOX9*^*flox/flox*^ mice is almost normal^15^. Indeed, the *Padi2*^−/−^ mice in our present study did not exhibit an obvious testicular phenotype.

In summary, we identified a novel testicular gene, *Padi2*, that is exclusively expressed in Sertoli cells and regulated by SOX9. Our *in vitro* data suggested that a possible function of PADI2 is to modify transcriptional regulation by SOX9. Further, during gonadal development, H3 is citrullinated in male and female gonads independent of *Padi2*. Although the role of the molecule in testicular development is likely limited because *Padi2*^−/−^ mice did not exhibit an obvious phenotype, our findings provide valuable insights that can be used to elucidate the molecular mechanisms of testicular development in mice.

## Materials and Methods

### Cell Culture

The murine testicular somatic cell line TM3 was obtained from the American Type Culture Collection (Manassas, VA, USA). HEK293T and TM3 cells were cultured in DMEM (Life Technologies, Carlsbad, CA, USA) supplemented with 10% fetal bovine serum (Invitrogen, Carlsbad, CA, USA) at 37 °C in 5% CO_2_.

### Mouse strains

C57BL/6J, hybrid F1 of C57BL/6J × DBA/2 (BDF1), and ICR mice were purchased from Sankyo Lab (Tokyo, Japan) and CLEA Japan (Tokyo, Japan). C57BL/6J-*Kit*^*W-v*^/J mice were provided by the RIKEN BRC through the National Bio-Resource Project of the MEXT, Japan. *Kit*^*Wv/*+^ mice were intercrossed to generate *Kit*^*Wv/Wv*^ embryos, which were depleted of germ cells. The generation of *Padi2*^−/−^ mice is explained in the next section.

Mouse embryos were collected from timed mating at noon of the day on which the mating plug was observed, designated as 0.5 d post-coitum (dpc). The genotype of the mouse embryos was determined by PCR assay. The generation of AMH-Cre:*Sox9*^*flox/flox*^ and *Foxl2*-null mice was reported previously^15,37^, and these mice were maintained in a mixed C57BL/6 background and C57B6/J/129/SVJ genetic background, respectively.

All animal protocols were approved by the Animal Care and Use Committee of the National Research Institute for Child Health and Development, Tokyo, Japan and the Center for Experimental Animals of Tokyo Medical and Dental University, Tokyo, Japan. All experiments were conducted in accordance with these approved animal protocols.

### Generation of *Padi2*^−/−^ mice by the CRISPR/Cas9 system

To disrupt exon 1 of *Padi2*, we designed a guide RNA (gRNA) using the online tool [https://crispr.dbcls.jp/]^38^ (Fig. 5a), and the selected sequence was 5′-CGGAATGCAGCCGCCTATACGG-3′. Construction of gRNA expression vectors, generation of template DNAs for *in vitro* transcription, and *in vitro* RNA synthesis were performed using a previously reported protocol^39^. The microinjection of mouse zygotes was performed as described previously^40,41^. Mouse zygotes were obtained by mating superovulated BDF1 females and BDF1 males. Genomic DNA samples from F0-generation offspring were extracted and tested for *Padi2* mutations by direct sequencing. We selected the line that carried a frameshift mutation (Fig. 5a), and it was backcrossed with C57BL/6J mice.

### Plasmid Construction

The Padi2 5′-UTR and intron 1 fragments were generated by PCR using genomic C57BL/6J DNA as a template and cloned into pCR2.1 using a TOPO-TA cloning kit (Invitrogen, Carlsbad, CA, USA). The fragments were cloned into the multiple cloning site of the pGL4.12 luciferase reporter vector (Promega, Madison, WI, USA) and named construct #1, construct #2, and construct #3 as shown in Fig. 3a. Construct #2, which had a mutation in the putative SOX9 response element (ACAAT > CGCGG) and FOXL2 response element (GTCAAGG > TGACCTT), was generated using Q5 DNA polymerase (New England Biolabs, Inc, Ipswich, MA, USA). Two forms of this construct were named construct #2 (mutant S2) and construct #2 (mutant F).

### Transfection and RNA interference(RNAi)

TM3 and HEK293T cells were transfected with expression vectors using Lipofectamine 2000 and 3000 (Invitrogen, Carlsbad, CA, USA) according to the manufacturer’s instructions. The total amount of DNA was standardized using the control vector, pcDNA3 (Invitrogen, Carlsbad, CA, USA).

TM3 cells were plated at 3.0 × 10^5^/well in 6-well plates 24 h before transfection. Two to four micrograms each of pSGSox9^42^ and pcDNA3Foxl2^43^ was added to each well and collected at 48 h post-transfection. For the RNA interference experiment, 4 µg of pSGSox9 and 50 µM *Padi2* siRNA oligo (**#:** 1320003; Life technologies, Carlsbad, CA, USA) were added to transfect TM3 cells plated at 1.25 × 10^5^ cells/well in a 6-well plate. The total amount of transfected siRNA oligo was standardized using the siRNA of LacZ.

HEK293T cells were plated at 1.5 × 10^5^ cells/well in a 12-well plate 24 h before transfection. The cells were transiently co-transfected with pGL4.12 constructs (250 ng; #E6671, Promega) and pRL-SV40 luciferase reporter plasmid (0.5 ng; #E2231, Promega) with expression plasmids (50–75 ng of pSGSox9 and 25–75 ng pcDNA3Foxl2).

### RNA isolation and Quantitative real time PCR (qRT-PCR)

Total RNA from TM3 cells was isolated using Isogen (Nippongene, Tokyo, Japan). Two to three µg of RNA from the cells was used as a template for the synthesis of cDNA using SuperScript III (Invitrogen, Carlsbad, CA, USA) and random primers (Invitrogen, Carlsbad, CA, USA) according to the manufacturer’s instructions. cDNA samples were diluted 1:4, and 1 μl was used in each 20 μl of qRT-PCR reaction, which contained SYBR Green PCR Master Mix (Roche, Basel, Switzerland). Transcript levels were analyzed by a previously described protocol^44^. Glyceraldehyde-3-phosphate dehydrogenase (*Gapdh*) was used as the normalizing gene to standardize qRT-PCR data.

Embryonic gonads from *Kit*^*Wv/Wv*^ mice and *Padi2*^−/−^ mice without mesonephros were dissected in PBS at the appropriate stages and immediately frozen in liquid nitrogen. Total RNA (60–300 ng) from embryonic gonads was isolated using Isogen (Nippongene) and was used for reverse transcription with SuperScript II (Invitrogen, Carlsbad, CA, USA) according to the manufacturer’s instructions. For quantitative PCR reactions, Power SYBR Master Mix (Applied Biosystems, Foster City, CA, USA) and SYBR Green PCR Master Mix (Roche, Basel, Switzerland) were used for *Kit*^*Wv/Wv*^ mice and *Padi2*^−/−^ mice, respectively. For *Kit*^*Wv/Wv*^ mice, transcript levels were analyzed on a 7900HT Fast Real-Time PCR System (Applied Biosystems) over 40 cycles of 95 °C for 10 s and 60 °C for 1 min, preceded by an initial 10-min step at 95 °C. For *Padi2*^−/−^ mice, an identical protocol as that used for TM3 cells was used for quantitative PCR reactions.

Gonadal samples from AMH-Cre:*Sox9*^*flox/flox*^ and *Foxl2*-null mice were analyzed by qRT-PCR as previously described^44^. All primers used were listed on Table 1.

**Table 1:** Primer List.

Name	Sequence (5′ to 3′)
**1**. **Primer for real-time PCR**	
Actb Fw	CGTGAAAAGATGACCCAG
Actb Rv	TGGTACGACCAGAGGCATACAG
Amh Fw	GCTAGTCCTACATCTGGCTG
Amh Rv	GGGTGTCCCGAGTAGGGCAG
Cyp26b1 Fw	TGACCATGCAGGAGCTGAAG
Cyp26b1 Rv	GCACAGCAGGGTGTTTTAGC
Gapdh Fw	CGTCCCGTAGACAAAATGGT
Gapdh Rv	TTGATGGCAACAATCTCCAC
Padi2 Fw	GTAGGCCACGTCGATGAGTT
Padi2 Rv	TCCCAGGCCCTTGAACATAAC
Ptgds Fw	TGGTTCCGGGAGAAGAAAGC
Ptgds Rv	TGGTTCCGGGAGAAGAAAGC
Sox9 Fw	AGTACCCGCATCTGCACAAC
Sox9 Rv	TACTTGTAATCGGGGTGGTCT
**2**. **Primer for** ***in situ*** **probe generation**	
Padi2 SP6 Fw1	**ATTTAGGTGACACTATAG**TCCTGTACCCCCAAGTCCTT^1^
Padi2 T7 Rv1	**TAATACGACTCACTATAGGG**GCCTGGATCTGGCTGTGTAG^2^
Padi2 SP6 Fw2	**ATTTAGGTGACACTATAGA**AGAGCCTGAGAAGCAGCCTA
Padi2 T7 Rv2	**TAATACGACTCACTATAGGG**TGTAATGGGATCTGACGCCC
Padi2 SP6 Fw3	**ATTTAGGTGACACTATAGA**GAACTTAGGACCTCTGGGCG
Padi2 T7 Rv3	**TAATACGACTCACTATAGGG**AACTGGTGTAGCCCAGAAGC

Bold characters indicate SP6 or T7 promoter sequence.

### Dual Luciferase assay

Transfected cells as described above were harvested and lysed 24 h after transfection, and both Firefly and Renilla luciferase activity was measured using a dual luciferase reporter assay system (#E1910, Promega). The firefly luminescence signal was normalized to that of Renilla, and the mean value of triplicated assay were calculated.

### *In situ* hybridization

To generate the probe for *Padi2*, part of the *Padi2* sequence was cloned into pCR2.1 (Invitrogen). PCR was performed with BIOTAQ (Bioline, London, UK), three sets of primers (Table 1), and the vector. Antisense digoxigenin-labeled RNA probes were generated by *in vitro* transcription using the PCR product, DIG RNA Labeling Mix (Roche), and ProbeQuant G-50 Micro Columns (GE Healthcare, Chicago, IL, USA) according to the manufacturer’s instructions. A probe for the sense strand of *Padi2* was used as a negative control.

Gonads and mesonephros from 13.5 dpc ICR mouse embryos were fixed overnight in 4% (w/v) paraformaldehyde with PBS at 4 °C and dehydrated with 25–75% methanol in PBS 0.1% (v/v) with Triton-X and embedded in 100% methanol. Subsequently, whole-mount *in situ* hybridization using the maleic acid buffer (MABT) method was performed as described previously^45^.

### Protein extraction and Western blotting

In order to confirm the abolished expression of PADI2 in null mice, tissue samples from the brains of adult mice were collected. The samples were eluted with RIPA buffer (50 mM Tris-HCl (pH 8.0), 150 mM NaCl, 0.1% SDS, 1% NP-40, 0.1% sodium deoxycholate) with cOmplete Protease Inhibitor Cocktail (#11697498001, Roche) and 0.1 M DTT. Subsequently, the samples were homogenized, sonicated, and centrifuged at 12,000 × *g* for 10 min at 4 °C. The supernatants were collected and subjected immunoblotting analysis performed with anti-β-actin antibody (#A1978; Sigma-Aldrich, St. Louis, MO, USA; 1:5000) and anti-PADI2 antibody (1:500, a kind gift from Dr. Takahara)^46^.

Histone extraction was performed following the manufacturer’s protocol (http://www.abcam.co.jp/protocols/histone-extraction-protocol-for-western-blot, Abcam, Cambridge, UK). Anti-H3 antibody (#ab1791; Abcam; 1:5000) and anti-histone H3 (citrulline R2 + R8 + R17) antibody (#5103; Abcam; 1:1000) were used for immunoblotting.

### Immunofluoresecense

Gonad and mesonephros complexes harvested from embryos at 13.5 dpc were fixed overnight in 4% (w/v) paraformaldehyde in PBS and embedded in paraffin. Antigen retrieval for paraffine sections (7 μm) was performed by microwave-boiling for 15 min in 0.01 M citrate buffer (pH 2.0). After three washes in PBS, the sections were incubated in 10% (w/v) FBS in PBS at room temperature for 60 min and subsequently incubated with a diluted primary antibody overnight at 4 °C. Anti-SOX9 antibody (AB5535; Millipore, Burlington, MA, USA) and anti-FOXL2 antibody (ab5096; Abcam) were diluted 1:500. After rinsing in PBS with 0.1% (v/v) Triton-X once and in PBS twice, sections were incubated with the secondary antibody conjugated with donkey anti-rabbit Alexa Fluor 488 (A21206, Life Technologies) and donkey anti-goat Cy3 (705–165–147; Jackson ImmunoResearch) for 1 h at room temperature. Sections were rinsed in PBS, counterstained with 4,6-diamidino-2-phenylindole (DAPI, Dojindo, 1:1000), and mounted using Fluoromount (Diagnostic BioSystems, Pleasanton, CA, USA). Digital images were taken using an Olympus FV-10i confocal laser-scanning microscope (OLYMPUS, Tokyo, JAPAN) and transferred to Photoshop CS (Adobe, San Jose, CA, USA) for the generation of figures.

### Search of the GEO Profiles database

The *Padi2* expression profile in fetal mouse gonads was obtained from the GEO Profiles database^12^ (GEO accession GSE27715^13^, GSE4818, and GSE5334 [Gaido K, Lehmann K *et al*., 2006]).

### Homology and binding sequence analysis

Phylogenetic analysis was performed on the whole human, mouse, pig, chicken, and zebrafish *Padi2* sequence. Each sequence was obtained from the National Center for Biotechnology Information (NCBI) reference sequence database (refseq) (https://www.ncbi.nlm.nih.gov/refseq/). These sequences were aligned using Molecular Evolutionary Genetics Analysis (MEGA; ver5; http://www.megasoftware.net/citations)^47^ and conserved SOX9 and FOXL2 binding sequences^31,48^ were identified.

### Statistical analysis

Student’s *t*-test was used to compare the control and the other groups. For comparison of multiple groups, ANOVA with Tukey’s method was employed.

## Electronic supplementary material

Supplementary Figure S1

## Data Availability

The datasets generated during and/or analyzed during the current study are available from the corresponding author on reasonable request. The sources of the datasets obtained from public repository are specified in each section above.
