# Supplementary material for: Peptidyl arginine deiminase 2 (Padi2) is expressed in Sertoli cells in a specific manner and regulated by SOX9 during testicular development
Source: Sci Rep. 2018 Sep 5;8:13263. doi: 10.1038/s41598-018-31376-8 (PMC6125343; doi:10.1038/s41598-018-31376-8)
Supplement: Supplementary file 1 — Supplementary Figure S1 [file 41598_2018_31376_MOESM1_ESM.pdf]

Supplementary Information

Peptidyl arginine deiminase 2 (*Padi2*) is expressed in Sertoli cells in a specific manner and regulated by SOX9 during testicular development

Atsumi Tsuji-Hosokawa, Kenichi Kashimada, Tomoko Kato, Yuya Ogawa, Risa Nomura, Kei Takasawa, Rowena Lavery, Andrea Coschiera, David Schlessinger, Vincent Harley, Shuji Takada, Tomohiro Morio

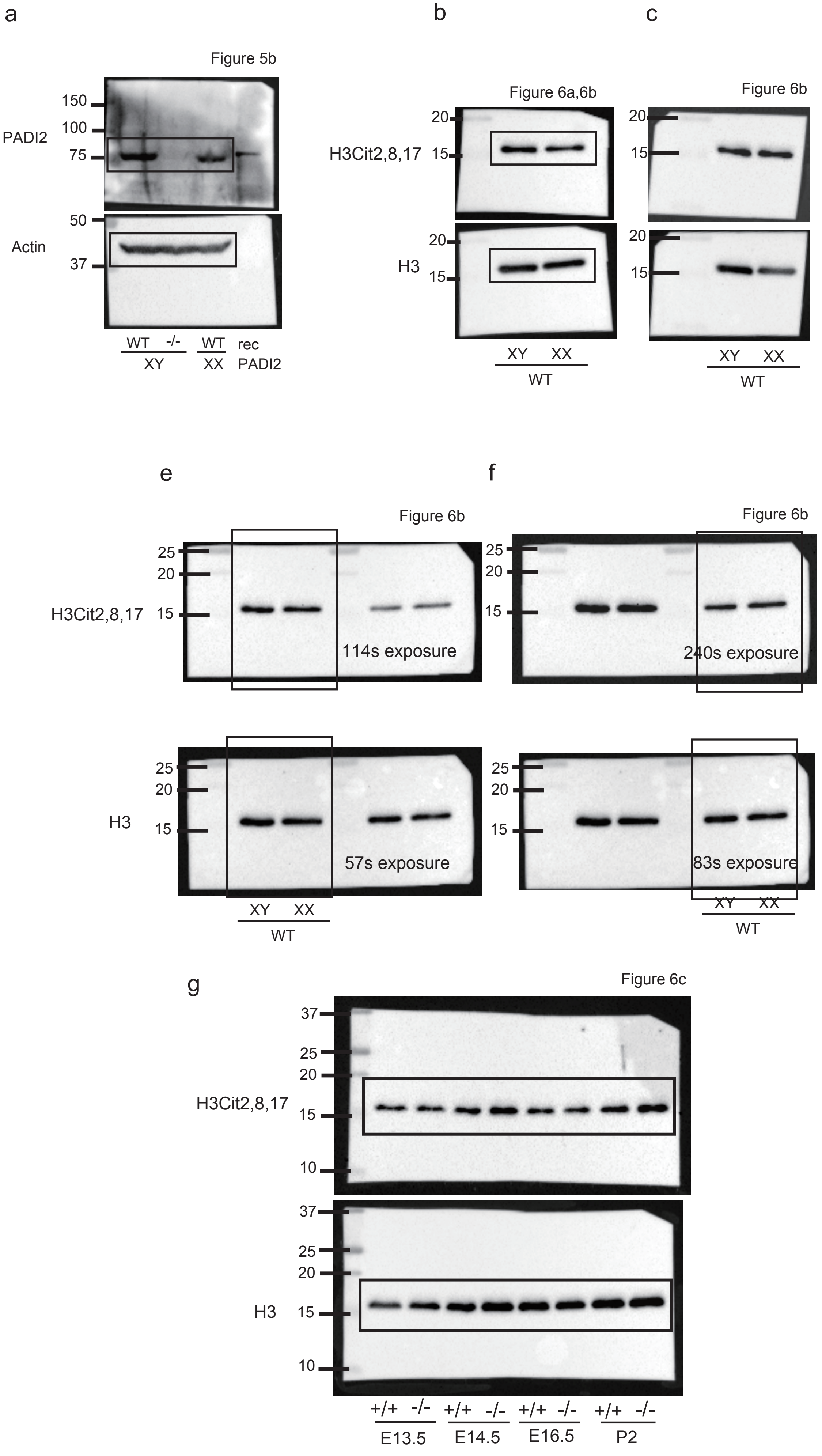
a
Figure 5b
150
100
75
PADI2
50
Actin
37
WT -/- WT rec
XY XX PADI2
b
Figure 6a,6b
20
H3Cit2,8,17
15
20
H3
15
XY XX
WT
c
Figure 6b
20
15
20
15
XY XX
WT
d
Figure 6b
25
20
15
25
20
15
XY XX
WT
e
Figure 6b
25
20
H3Cit2,8,17 15
114s exposure
25
20
H3
15
57s exposure
XY XX
WT
f
Figure 6b
25
20
15
240s exposure
25
20
15
83s exposure
XY XX
WT
g
Figure 6c
37
25
20
H3Cit2,8,17
15
10
37
25
20
H3
15
10
+/+ -/- +/+ -/- +/+ -/- +/+ -/-
E13.5 E14.5 E16.5 P2

Figure S1

Figure Legend

Supplementary Figure1 : The full sets of data images of immunoblotting analyses for Figure 5 and 6

a : The full size images of Figure 5a. The molecular weights of PADI2 and Actin were 76kDa and 42kDa, respectively. Recombinant PADI2 was used as a positive control. The open squares indicate the trimmed area used in Figure 5a. **b** : The full size images of Figure 6a. The molecular weights of H3Cit2,8,17 and H3 and were 17kDa. Based on the data, quantitative analysis in Figure 6b was carried out. **c-f** : The full size images used for quantitative analysis of Figure 6b. The images of Figure S1e and S1f were obtained from the same membrane with different exposure time. The bands in the opened squares were used for the statistical analysis. **g** : The full size images of Figure 6c, and the open squares indicate the trimmed area used in Figure 6c. The molecular weights of H3Cit2,8,17 and H3 and were 17kDa.
